# Supplementary material for: Parallel analysis of tri-molecular biosynthesis with cell identity and function in single cells
Source: Nat Commun. 2019 Mar 12;10:1185. doi: 10.1038/s41467-019-09128-7 (PMC6414513; doi:10.1038/s41467-019-09128-7)
Supplement: Supplementary file 1 — Supplementary Information [file 41467_2019_9128_MOESM1_ESM.pdf]

Supplementary material for**:**

Kimmey, et al., Parallel Analysis of Tri-molecular Biosynthesis with Cell Identity and Function in Single Cells

Contents (in order of appearance):

1. Supplementary Table 1-6
2. Supplementary Figure 1-6
3. Supplementary References

Kimmey, Supplementary Table 1. Mass-cytometry antibody staining panel for HeLa cell-cycle SOM3B analysis

Metal	Mass	Antibody Target	Staining uL	Concentration (Stock, ug/mL)	Concentration (Stain, ug/mL)	Manufacturer	Clone	Catalog #	Cell Target
In	115	A2B5	2.00	100	2.00	Thermo	105	130-093-394	
I	127	IdU	-	-	-	-	-		DNA Synthesis
Nd	142	Active_Caspase3	1.00	200	2.00	BD	C92-605	559565	Apoptotic cells
Nd	143	p4EBP1_Thr37/T46	2.00	200	4.00	CST	236B4	custom, 2855	mTOR/AKT pathway
Sm	152	pAKT_Thr308	2.00	50	1.00	BD	J1-223.371	558316	mTOR/AKT pathway
Gd	156	PBX1	3.00	100	3.00	CST	Polyclonal	custom, 4342	Intranuclear transcription factor
Gd	158	Puromycin	0.75	100	0.75	MilliporeSigma	12D10	MABE343	Protein synthesis
Dy	162	Cyclin B1	2.00	200	4.00	BD	GNS-1	554177	G2 phase
Ho	165	rRNA	1.00	400	4.00	Novus	Y10b	NB100-662	Ribosomal RNA
Yb	171	cPARP	1.00	200	2.00	BD	Asp214	552597	Apoptotic cells
Yb	172	pS6_Ser235/236	2.00	200	4.00	BD	N7-548	custom	mTOR/AKT pathway
Lu	175	BRdU	2.00	200	4.00	BD	3D4	BDB555627	RNA Synthesis
Yb	176	p-HH3-Ser28	0.75	200	1.50	BioLegend	HTA28	641002	Mitotic cells
Ir	191	DNA Intercalator	-	-	-	-	-		DNA
Pt	195	Cisplatin	-	-	-	-	-		Viability

= Extracellular
= Intracellular
= Non-antibody parameter

Kimmey, Supplementary Table 2. Mass-cytometry antibody staining panel for SOM3B analysis on *ex vivo* stimulated human whole blood monocluear cells

Metal	Mass	SPADE	Antibody Target	Staining uL	Concentration (Stock, ug/mL)	Concentration (Stain, ug/mL)	Manufacturer	Clone	Catalog #	Cell Target
In	113	No	CD235ab	0.50	200	1.00	BioLegend	HIR2	306602	Erythroid cell
In	115	No	CD66	1.00	200	0.30	BD	B1.1/CD66	551354	Granulocyte
I	127	No	IdU	-	-	-	-	-		DNA Synthesis
La	139	No	CD61	0.25	200	0.50	BioLegend	VI-PL2	336402	Platelet
Ce	140	No	IgM	0.50	400	2.00	Invitrogen	Polyclonal	H15000	B cell
Pr	141	Yes	CD7	1.00	200	2.00	BD	M-T701	555359	T/NK cell
Nd	142	No	Active_Caspase3	1.00	100	1.00	BD	C92-605	559565	Apoptotic cells
Nd	143	No	p4EBP1_Thr37/46	1.00	350	3.50	CST	236B4	custom, 2855	mTOR/AKT pathway
Nd	145	No	p-p38	2.00	100	2.00	BD	36/p38	612288	Signaling
Nd	148	No	CD16	1.50	100	1.50	Fluidigm	3G8	3148004B	NK/Monocytes
Sm	149	No	CD69	1.00	75	0.75	BD	FN50	555529	Activation marker
Nd	150	Yes	CD56	0.50	200	1.00	BD	NCAM16.2	559043	NK cell
Eu	151	Yes	CD123	0.50	200	1.00	BioLegend	6H6	306002	Dendridic cell
Sm	152	No	TNFalpha	1.00	100	1.00	Fluidigm	Mab11	3152002B	Cytokine
Eu	153	No	pSTAT1_Tyr701	2.00	100	2.00	BD	4A	custom	Signaling
Sm	154	Yes	CD45	1.00	75	0.75	BioLegend	HI30	304002	Pan immune cell marker
Gd	155	No	CD45RA	0.50	100	0.50	Fluidigm	HI100	3155011B	Pan marker/Naive T Cell
Gd	157	Yes	CD4	1.00	400	4.00	BioLegend	RPA-T4	300502	T cell subset
Gd	158	No	Puromycin	1.00	100	1.00	MilliporeSigma	12D10	MABE343	Protein synthesis
Tb	159	Yes	CD20	1.00	200	2.00	BD	2H7	302302	B cell
Dy	161	Yes	CD14	1.00	200	2.00	BD	M5E2	301802	Monocyte
Ho	165	No	rRNA	0.50	400	2.00	Novus	Y10b	NB100-662	Ribosomal RNA
Er	166	Yes	CD19	0.30	500	1.00	BioLegend	HIB19	302202	B cell
Er	167	Yes	CD38	0.20	200	0.25	BioLegend	HIT2	303502	Plasma cell
Er	168	No	IFNgamma	1.00	100	1.00	Fluidigm	B27	3168005B	Cytokine
Er	170	Yes	CD3	1.00	50	0.50	BD	UCHT1	555329	Pan T cell
Yb	171	No	pZAP70	1.00	100	1.00	Fluidigm	17a	3171005A	Signaling
Yb	172	No	pS6_Ser235/236	1.00	200	2.00	BD	N7-548	custom	mTOR/AKT pathway
Yb	173	Yes	CD11c	1.00	200	2.00	BioLegend	Bu15	337202	Dendridic cell
Lu	175	No	BRdU	2.00	200	4.00	BD	3D4	BDB555627	RNA Synthesis
Ir	191	No	DNA Intercalator	-	-	-	-	-		DNA
Pt	195	No	Cisplatin	-	-	-	-	-		Viability
Bi	209	Yes	HLA-DR	0.50	200	1.00	BioLegend	L243	307602	MHC class II

= Extracellular (blue)
= Intracellular (orange)
= Non-antibody parameter (grey)

Kimmey, Supplementary Table 3. Evidence for manual annotation of whole blood mononuclear cell node groups in SPADE maps

Group Name	Reference	Evidence For	Note
Platelets	3, 7, 8	CD61+ CD45low	Used for *in silico* mononuclear cell purification
Red Blood Cells	3, 7	CD235ab+ CD45mid-low	Used for *in silico* mononuclear cell purification
Granulocytes	2, 3	CD66+ CD45low	Used for *in silico* mononuclear cell purification
CD4+ T Cell	6, 7	CD3+ CD4+ CD19- HLADR-	
CD4neg T Cells	6, 7	CD3+ CD4neg CD19- HLADR-	CD8+ T cells
B Cells	3, 6, 7	CD20+ CD19+ HLADR+	
NK	6, 7	CD56++ CD38het CD16het CD14- CD19- CD20-	
CD16+ Monocyte	6	CD14low CD16+	
CD14+ Monocyte	6	CD14+ CD16neg	
cDC	6	CD11c+ HLADR+ CD14-	
pDC	6, 7	CD123+ HLADR+ CD14-	
Basophil	1	CD38+ CD123+ CD19- HLADR-	

Kimmey, Supplementary Table 4. Mass-cytometry antibody staining panel for SOM3B analysis on the healthy human hematopoietic continuum *ex vivo*

Metal	Mass	SPADE	Wanderlust	Antibody Target	Staining uL	Concentration (Stock, ug/mL)	Concentration (Stain, ug/mL)	Manufacturer	Clone	Catalog #	Target Description
Y	89	Yes	No	CD45	0.80	500	4.00	BioLegend	2D1	3089003B	Pan immune cell marker
In	113	Yes	No	CD235ab	1.00	200	2.00	BioLegend	HIR2	306602	Erythroid cell
In	115	Yes	No	CD71	1.50	200	3.00	BioLegend	CY1G4	334102	Erythroid cell
I	127	No	No	IdU	-	-	-	-	-		DNA Synthesis
La	139	Yes	No	CD61	0.50	200	0.25	BioLegend	VI-PL2	336402	Megakaryocyte/platelet
Ce	140	Yes	Yes	IgM	0.50	400	2.00	Invitrogen	Polyclonal	H15000	B cell
Pr	141	Yes	No	CD7	1.00	200	2.00	BD	M-T701	555359	T & NK cell
Nd	142	No	No	Active_Caspase3	1.50	100	1.50	BD	C92-605	559565	Apoptotic cells
Nd	143	Yes	Yes	CD179b	2.00	200	4.00	BioLegend	HSL11	349802	B cell progenitor
Nd	144	Yes	No	CD11b	4.00	100	4.00	Fluidigm	ICRF44	3209003B	Myeloid
Nd	145	Yes	No	CD4	2.00	100	2.00	Fluidigm	RPA-T4	3145001B	T cell subset
Nd	146	Yes	No	CD8	1.00	100	1.00	Fluidigm	RPA-T8	3146001B	T cell subset
Sm	147	Yes	No	CD11c	2.00	100	2.00	Fluidigm	Bu15	3147008B	Dendridic cell
Nd	148	Yes	Yes	CD34	2.00	200	4.00	BioLegend	581	343502	Hematopoeit stem cell & progenitors
Sm	149	Yes	Yes	CD179a	1.00	150	1.50	BioLegend	HSL96	347402	B cell progenitor
Nd	150	No	No	GATA3	2.00	200	4.00	BD	L50-823	558686	Intracellular Transcription Factor
Eu	151	Yes	No	CD123	2.00	100	2.00	Fluidigm	6H6	306002	Dendridic cell
Sm	152	Yes	No	CD10	3.00	200	6.00	BioLegend	HI10a	312202	B cell progenitor
Eu	153	Yes	Yes	CD19	0.50	500	2.50	BioLegend	SJ25C1	363002	B cell
Sm	154	Yes	No	CD56	0.33	200	0.75	BD	NCAM16.2	559043	NK cell
Gd	155	Yes	No	CD45RA	2.00	100	2.00	Fluidigm	HI100	3155011B	Pan marker/Naive T Cell
Gd	156	Yes	No	CD14	1.00	100	1.00	BioLegend	M5E2	301802	Monocyte
Gd	157	Yes	No	CD66	1.00	200	2.00	BD	B1.1	551354	Granulocyte
Gd	158	No	No	Puromycin	1.00	100	1.00	MilliporeSigma	12D10	MABE343	Protein synthesis
Tb	159	Yes	No	Biotin (CD90-Biotin*)	1 (0.5)	50 (500)	0.50 (2.50)	BioLegend (BD)	1D4-C5 (5E10)	409002 (555594)	Hematopoeit stem cell & progenitors
Gd	160	Yes	Yes	CD24	1.00	200	2.00	BioLegend	ML5	311102	B cell progenitor
Dy	161	Yes	Yes	CD20	1.00	200	2.00	BD	2H7	555621	Immature B cell
Dy	162	Yes	No	CD127	3.00	200	6.00	BioLegend	HCD127	317602	T cells (IL7Ralpha)
Dy	163	Yes	No	CD36	0.50	400	2.00	BioLegend	5-271	336208	Platelets & erythroid cell
Dy	164	Yes	Yes	TdT	1.50	200	3.00	BD	E17-1519	custom	Terminal transferase enzyme
Ho	165	No	No	rRNA	1.00	400	4.00	Novus	Y10B	NB100-662	Ribosomal RNA
Er	166	No	No	FOXP1	0.50	300	1.50	CST	D35D10	4402BF	Intracellular Transcription Factor
Er	167	No	No	Ki67	1.00	200	2.00	BD	B56	3168022D	Intracellular Mitotic Factor
Er	168	Yes	Yes	CD38	1.50	200	3.00	BioLegend	HIT2	303502	Hematopoietic progenitor/plasma cell
Tm	169	No	No	CD25	1.00	200	2.00	BioLegend	BC96	302602	IL2Ralpha chain
Er	170	Yes	No	CD3	2.00	50	1.00	Fluidigm	UCHT1	555329	Pan T cell
Yb	171	Yes	No	CD117	2.00	100	2.00	BioLegend	104D2	313202	Hematopoietic rogenitor
Yb	172	No	No	pS6_Ser235/236	1.00	100	1.00	BD	N7-548	custom	mTOR/AKT pathway
Yb	173	Yes	No	CD135	1.50	100	1.50	BioLegend	BV10A4H2	313302	FMS like tyrosine kinase (FLT3)
Yb	174	Yes	No	CD2	1.00	200	2.00	BioLegend	TS1/8	309202	T cells
Lu	175	No	No	BRdU	2.00	200	4.00	BD	3D4	BDB555627	RNA Synthesis
Yb	176	No	No	SATB1	1.50	200	3.00	Sigma	SAT-5	SAB4200481	Intracellular Transcription Factor
Ir	191	No	No	DNA Intercalator	-	-	-	-	-		DNA
Pt	195	No	No	Cisplatin	-	-	-	-	-		Viability
Bi	209	Yes	No	HLA-DR	1.50	200	3.00	BioLegend	L243	307602	MHC class II

= Extracellular
= Intracellular
= Non-antibody parameter
* anti-CD90-Biotin antibody included in extracellular stain, anti-Biotin included in intracellular stain.
(anti-CD90 reagent information in parentheses)

Kimmey, Supplementary Table 5. Evidence for manual annotation of bone marrow mononuclear cell node groups in SPADE maps

Group Name	Reference	Phenotype
HSC	5, 7	CD34++ CD90++ CD45low CD38neg
Early Progenitor (EP)	5, 7	CD34++ CD90++ CD45low CD38+
Intermediate Progenitor (IP)	5, 7	CD34mid CD38+
Late Progenitor (LP)	5, 7	CD34+/- CD38+ TdT-het
GMP	4, 7	CD34+ CD38+ CD123+(low) CD45RA+
MEP	3, 4, 7	CD34+ CD38+ CD123neg CD45RAneg
Pro Erythroblast	2, 3, 7	CD34het CD36+ CD38+ CD71+ CD45RAneg CD123neg CD235ab-neg
Basophilic Erythroblast	3, 7	CD38het CD36++ CD71+ CD34neg CD45RAneg CD123neg CD235ab-neg
Polychromatophilic Erythroblast	3, 7	CD36++ CD71+ CD235ab+ CD34neg CD38neg CD45RAneg CD123neg
Pro B	2, 7	TdT+ CD34+ CD38+ CD179a+ CD179b+ CD19neg CD24neg
Pre B I	2, 7	TdT+ CD34het CD38+ CD24+ CD19low CD179a+ CD179b+
Pre B II	2, 7	CD19+ CD20het IgM+ CD38+ CD179a+ CD179b+ CD34neg
Early Immature B (E-iB)	2, 7	CD19+ CD20het IgM+ CD38+ CD179a-neg CD179b-neg
Immature B (iB)	2, 7	CD19+ CD20+ IgM+
Plasma Cells	9, 2, 6	CD19+ CD38+++ CD45low CD20neg
Monoblast (Mb)	2, 3, 7	CD38+ HLADR+ CD45RA+ CD123+ CD11c+
Promonocyte	2, 3, 7	HLADR+ CD11b+ CD36+ CD45+ CD14low
Monocyte	2, 3	HLADR+ CD11b++ CD36+++ CD45+ CD14+
Myeloid	7	CD14het CD36+ HLADRhet
Platelet	8	CD61++, CD36++, CD14neg
cDCs	6	HLADR+ CD11c+ CD123neg CD14neg
Pre pDCs	7	HLADR+ CD11b+ CD36++ CD45+ CD38high
pDCs	6, 7	HLADR+ CD11b+ CD36++ CD45+ CD38low
Basophils (Baso)	1	CD123++ CD38+ HLA-DRneg CD11bneg
Promyelo/myelocyte (P/M)	2, 3	CD66mid/high CD38+ HLADR
Neutrophils	2, 3	CD66+ HLADRneg
NK	6, 7	CD7+ CD56+ CD3neg
NKT	7, 8	CD3+ CD7+ CD56het CD4neg CD8neg CD45RAneg
Double Negative T	10	CD3+ CD45RA+ CD4neg, CD8neg
CD8+ Naïve (CD8+N)	6, 7	CD3+ CD8a+ CD45RA+
CD8+ Memory (CD8+M)	6, 7	CD3+ CD8a+ CD45RAneg
CD4+ Naïve (CD4+N)	6, 7	CD3+ CD4+ CD45RA+
CD4+ Memory (CD4+M)	6, 7	CD3+ CD4+ CD45RAneg

Kimmey, Supplementary Table 6. Raw cell event counts and p-values for bone marrow mononuclear cell phenotypic clusters

Phenotypic Cluster	Figure 5 - Donor 1 Raw Cell Number	Figure 5 - Donor 1 BRU & Puromycin vs HSC (Friedman-Rafsky p-value)	Figure 5 - Donor 1 *p* symbol	Figure S6 - Donor 2 Raw Cell Number	Figure S6 - Donor 2 BRU & Puromycin vs HSC (Friedman-Rafsky p-value)	Figure S6 - Donor 2 *p* symbol
HSC	1921	0.611741884		1334	0.664898499	
Early Progenitor (EP)	4152	3.38E-07		3014	4.88E-09	*
Intermediate Progenitor (IP)	406	5.95E-17	**	257	1.95E-15	**
Late Progenitor (LP)	413	9.18E-17	**	256	2.27E-13	*
GMP	781	4.16E-28	***	366	1.30E-31	****
MEP	951	1.12E-04		975	3.53E-05	
Pro Erythroblast	600	6.02E-23	***	223	5.90E-23	***
Basophilic Erythroblast	1155	2.43E-32	****	610	1.32E-31	****
Polychromatophilic Erythroblast	1830	1.84E-25	***	2379	1.21E-19	**
Monoblast (Mb)	4285	4.47E-40	****	1202	4.00E-37	****
Promonocyte	672	9.79E-42	****	45	1.37E-43	****
Monocyte	18873	4.59E-33	****	3860	4.25E-26	***
Myeloid	68831	9.23E-27	***	28198	3.76E-21	**
cDCs	6079	6.09E-23	***	5820	8.35E-18	**
Platelet	5919	1.36E-31	****	3543	9.40E-17	**
Promyelo/myelocyte (P/M)	6448	7.13E-37	****	2288	9.31E-27	***
Neutrophils	152761	2.50E-35	****	80709	2.50E-22	***
Pre pDCs	5304	1.30E-31	****	4128	3.46E-24	***
pDCs	4707	1.88E-25	***	2671	5.33E-14	*
Basophils (Baso)	3360	0.044257778		2025	0.044280291	
Double Negative T	6481	7.23E-04		1718	0.144161372	
CD4+ Naïve (CD4+N)	38811	0.001439377		37930	0.011583733	
CD4+ Memory (CD4+M)	54667	0.001435782		33170	0.03251306	
CD8+ Naïve (CD8+N)	55446	0.011573134		35609	1.41E-06	
CD8+ Memory (CD8+M)	65202	0.003502373		32347	0.059233529	
NKT	20754	8.97E-04		3869	0.016618434	
NK	51667	6.48E-13	*	35749	1.56E-08	*
Pro B	297	8.99E-15	**	343	2.94E-15	**
Pre B I	919	2.49E-07		439	5.40E-06	
Pre B II	4334	3.68E-21	**	725	1.92E-19	**
Early Immature B (E-iB)	13071	0.00288132		3947	6.34E-05	
Immature B (iB)	43751	0.016628616		16977	8.97E-04	
Plasma Cells	2882	1.17E-37	****	441	7.04E-31	****

p-value symbol key	
>1E-7	no symbol
1E-7 to 1E-14	*
1E-14 to 1E-21	**
1E-21 to 1E-28	***
< 1E-28	****

Kimmey, Supplementary Figure 1

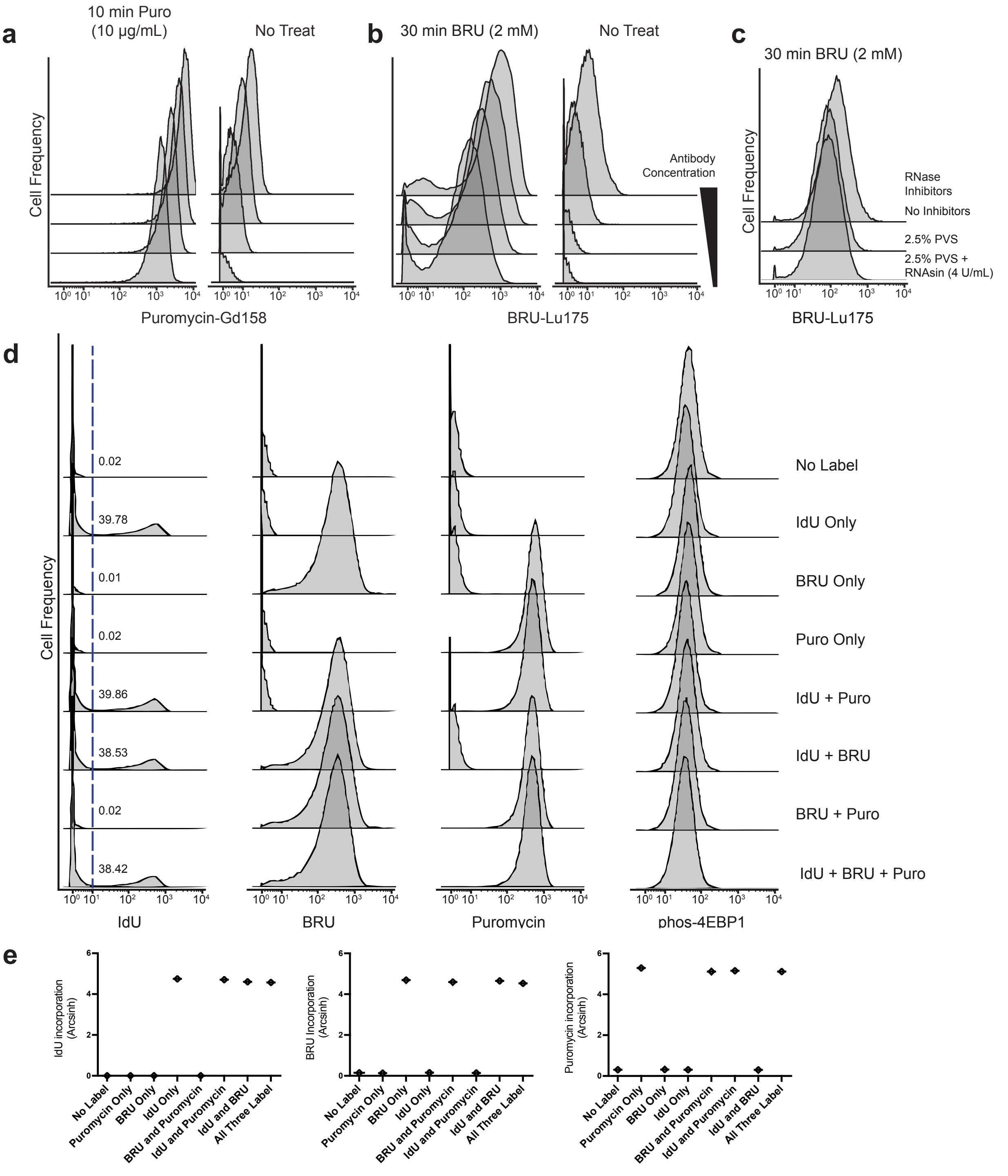

f

Friedman-Rafsky p-value

Conditions		IdU and p4EBP1*	BRU and p4EBP1	Puromycin and p4EBP1
All Three Label vs:	No Label	3.14259E-34	1.42849E-42	2.74228E-44
	Puromycin Only	1.90175E-30	1.42849E-42	0.238951
	BRU Only	2.12653E-38	0.197216	2.74228E-44
	IdU Only	0.388260	5.38E-42	2.74228E-44
	BRU and Puromycin	5.46345E-32	0.443564	0.443569
	IdU and Puromycin	0.500000	1.44225E-42	0.388257
	IdU and BRU	0.388227	0.388248	2.76983E-44
	All Three Label	0.500000	0.500000	0.500000

Supplementary Figure 1: Validation of SOM3B labeling reagents. **a** Human embryonic kidney 293T (HEK 293T) cells labeled with puromycin (left) and untreated (right), stained with increasing concentration of isotope conjugated anti-puromycin antibody. **b** HeLa cells labeled with BRU (left) and untreated (right), stained with increasing concentration of anti-BRdU antibody (concentrations; 1, 2, 4, 8 μg/mL). **c** Histograms of detected BRU in HeLa cells stained with antibody in the presence or absence of RNase inhibitors. **d** Analysis of Jurkat cells labeled with one or combinations of SOM_3B labeling reagents. **e** Quantification of bootstrapped median values for data in **d**, error bars indicate 95 percent bootstrap confidence interval. **f** Freidman-Rafsky probability values for data in **c** and **d** (*45-48k single cells for IdUpostive evaluations [see methods], 115-120k single cell events for BRU and puromycin evaluations). Experiments were performed multiple times, representative plots displayed in **a**, **b**, and **c** with 40,000 to 100,000+ single cell events per condition.

Kimmey, Supplementary Figure 2

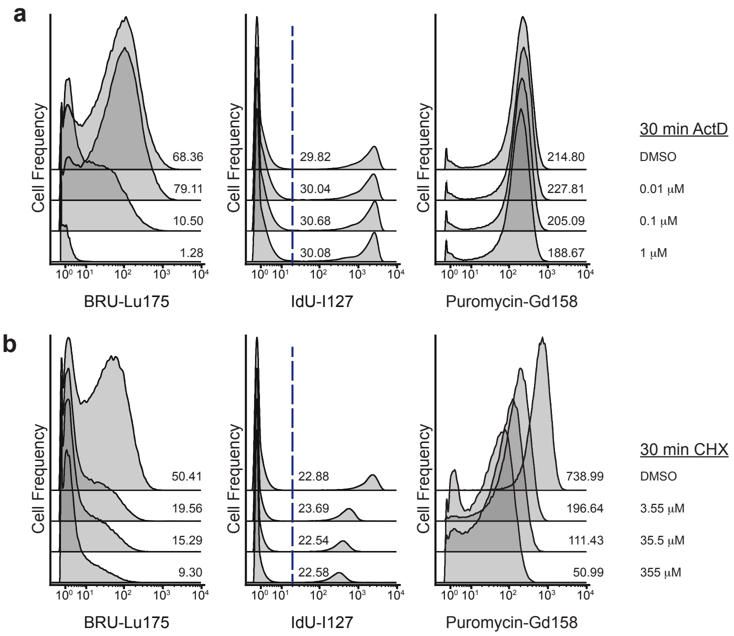

Supplementary Figure 2: Dose-dependent block of BRU and puromycin incorporation with specific transcription and translation inhibitors. a SOM_3B analyses in HeLa cells treated with increasing concentration of active RNA synthesis inhibitor Actinomycin D before labeling. **b** SOM_3B analyses in HeLa cells treated with increasing concentration of active translation inhibitor Cycloheximide before labeling. Inlaid histogram values are medians for BRU and puromycin incorporation, and frequency of cells positive for IdU incorporation. Experiments were performed multiple times, representative plots displayed with 100,000+ single cell events per condition.

Kimmey, Supplementary Figure 3

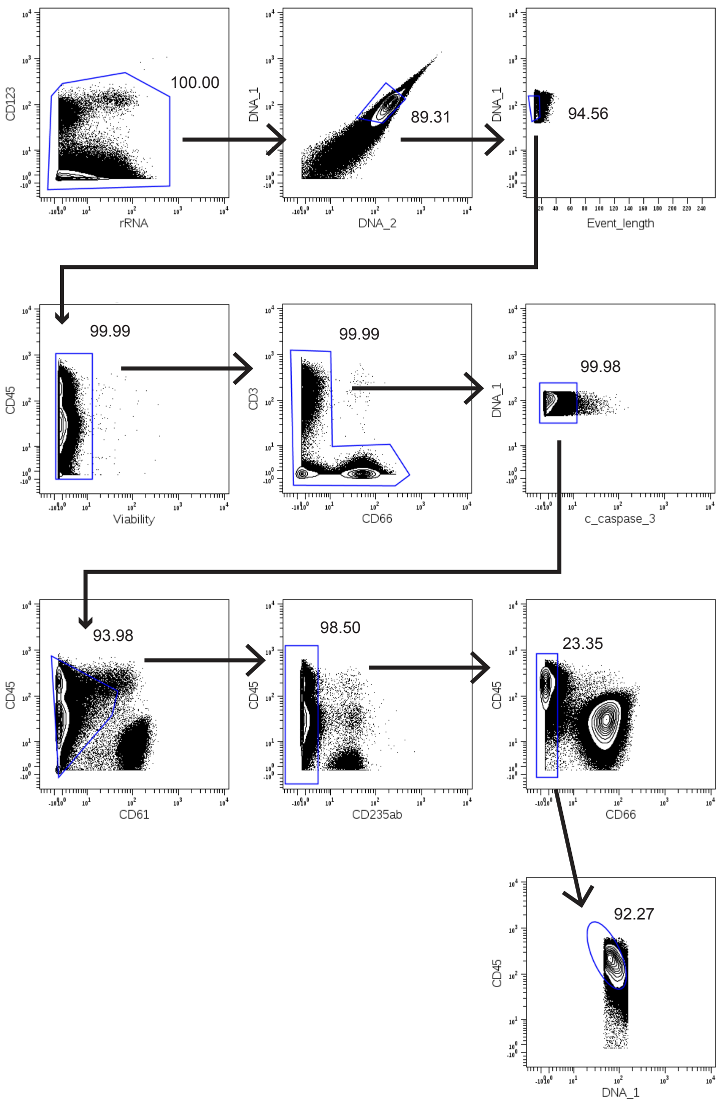

Supplementary Figure 3: Gating scheme to isolate peripheral immune mononuclear cells *in silico*. Mass-cytometry gating scheme to isolate mononuclear cell events from whole blood prior to phenotypic clustering with by SPADE. Doublets, debris, non-viable (cisplatin positive), and non-apoptotic (c[leaved]_caspase_3 positive) events were removed. Mononuclear cells are isolated by removal of platelets (CD61), red blood cells (CD235ab), and granulocytes (CD66, see Supplementary Table 3). Arrows indicate gating hierarchy.

Kimmey, Supplementary Figure 4

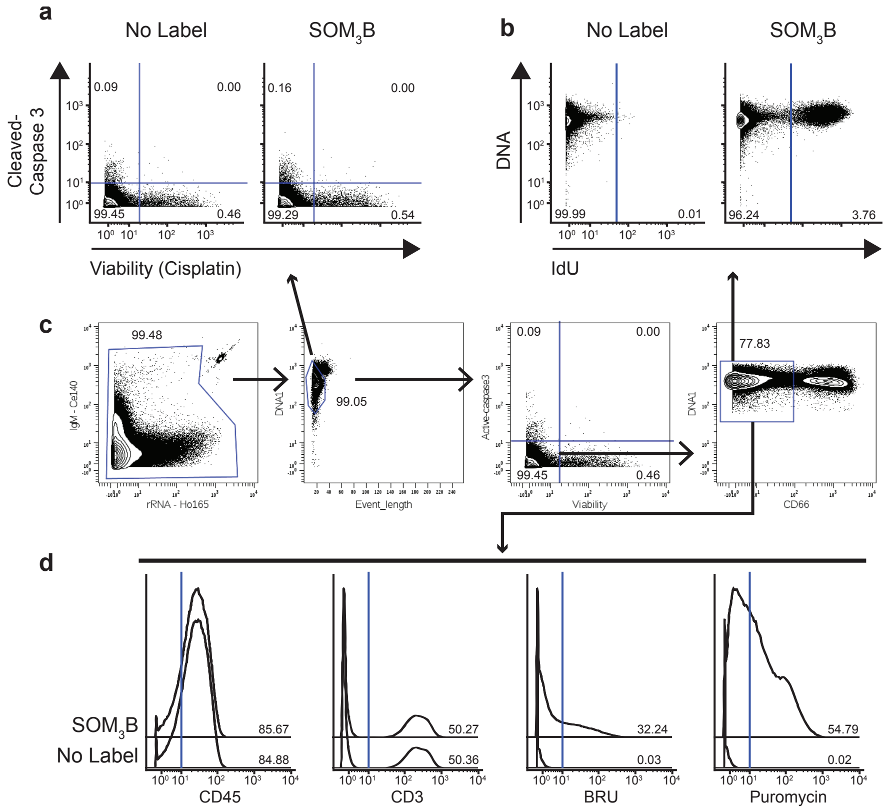

Supplemental Figure 4: Bone marrow cell fitness and surface protein expression are unperturbed by labeling with SOM3B reagents. **a** Biaxial plots with inlaid frequency of bone marrow cells undergoing apoptosis (cleaved-Caspase3 positive) and non-viable cells (positive cisplatin staining) when unlabeled or labeled with SOM3B reagents. **b** Frequency of bone marrow cells positive for IdU incorporation after labeling with SOM3B reagents. **c** *In silico* gating scheme for plots in panel **a**, **b**, and **d**. **d** Histograms of surface protein expression and SOM3B parameters in unlabeled and labeled bone marrow cells. Inlaid quadrant numbers indicate the percent of total events within each gate, 300,000+ single cell events per condition.

Kimmey, Supplementary Figure 5

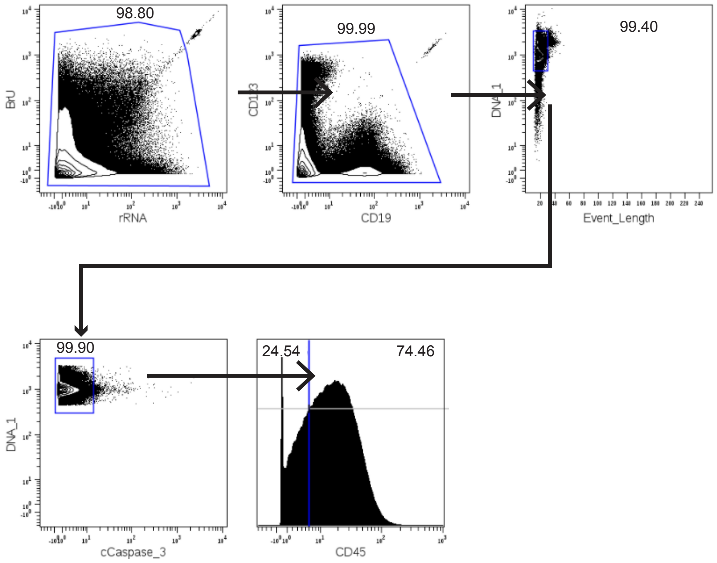

Supplemental Figure 5: Gating scheme to isolate bone marrow mononuclear immune cells *in silico*. Mass-cytometry gating scheme to isolate CD45 positive, mononuclear bone marrow cell events for adult donors before phenotypic clustering with SPADE and developmental progression analysis with Wanderlust. Arrows indicate gating hierarchy.

Kimmey, Supplementary Figure 6

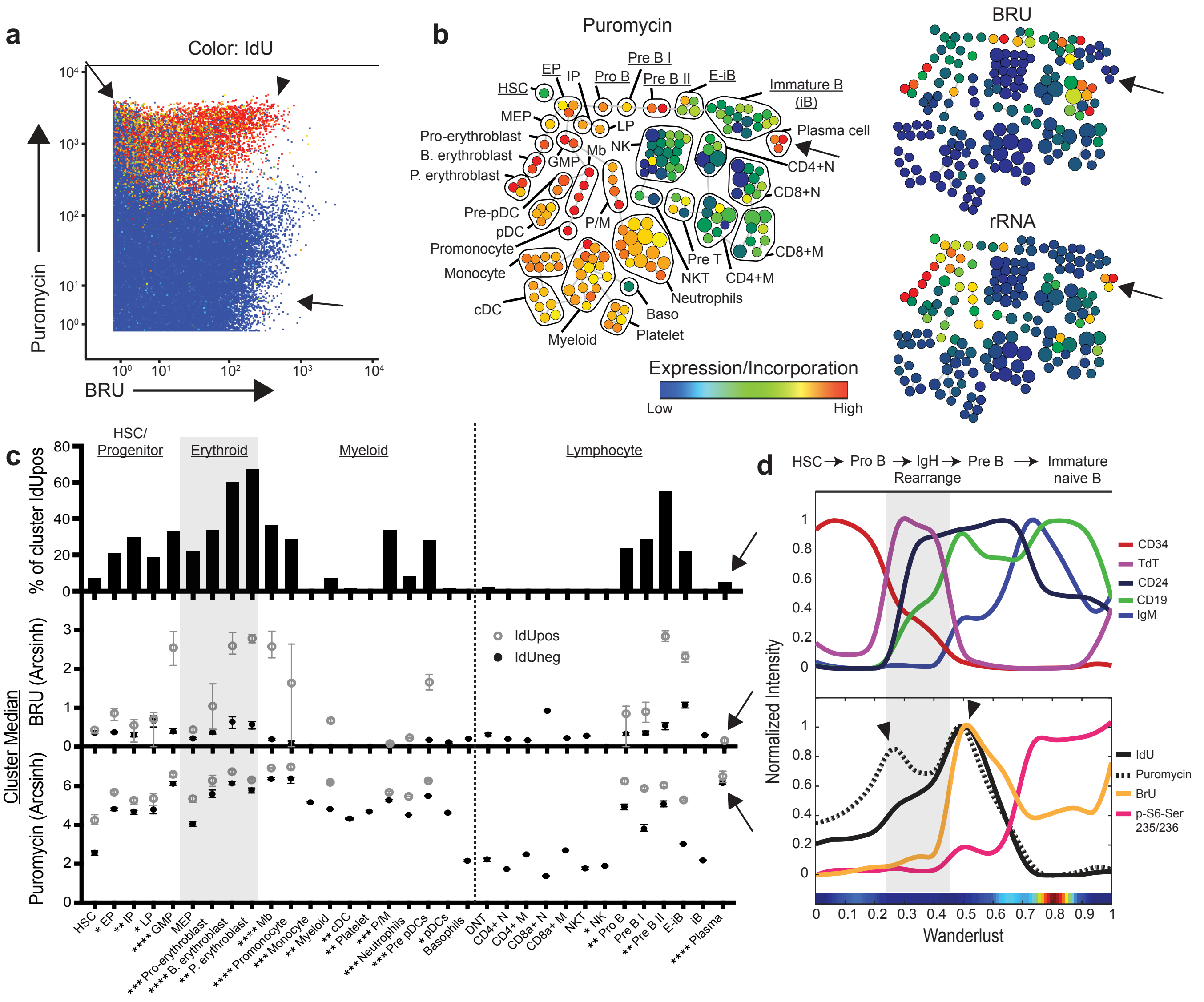

Supplemental Figure 6: Dynamic DNA, RNA, and protein synthesis across a healthy human hematopoietic continuum can be linked to cellular function and developmental identity (healthy human donor #2). **a** Plot of CD45pos immune cells from a second healthy human bone marrow donor (color overlaid: IdU incorporation, color range: 0, 410 ion counts). Arrows highlight cells with high activity of RNA or protein synthesis, arrowhead indicates high biosynthesis activity of all three biomolecules. **b** Manually annotated SPADE map of single cell data in **a**, clustered according to immune-cell phenotype and color-overlaid by the median incorporated puromycin (left), BRU (top right) and ribosomal RNA expression (bottom right) for each cluster. **c** Quantification of DNA synthesis (percent IdU positive), RNA and protein synthesis for each phenotype group in **b**. Values are displayed for both IdU positive and IdU negative sub-populations only when greater than 4% of the parent population is positive for IdU. Error bars, 95 percent bootstrap confidence interval, see Supplementary Table 6 for total cell observations in each phenotypic group. **d** Wanderlust trajectory analysis of B-cell lineage (underlined in **b**) ordered according to developmental pseudo-time with functional markers (top), and activity of DNA, RNA, protein synthesis, and p-S6 expression in the same B-cell developmental axis (bottom). Arrowheads highlight developmental stages immediately before, and after, B-cell somatic recombination. Experiment was performed on two independent donors, with additional donor data presented in Figure 5. p-values are expressed as follows: $1.0\text{x}10^{-7} > p > 1.0\text{x}10^{-14}$*; $1.0\text{x}10^{-14} > p > 1.0\text{x}10^{-21}$**; $1.0\ \text{x}10^{-21} > p > 1.0\text{x}10^{-28}$***; $p < 1.0\text{x}10^{-28}$****. Significance was

obtained by the Friedman-Rafsky test, evaluating BRU and puromycin incorporation in a given phenotypic population relative to HSC.

Supplementary References

1. Han, X. *et al.* Immunophenotypic study of basophils by multiparameter flow cytometry. *Arch. Pathol. Lab. Med.* **132,** 813–819 (2008).
2. van Lochem, E. G. *et al.* Immunophenotypic differentiation patterns of normal hematopoiesis in human bone marrow: reference patterns for age-related changes and disease-induced shifts. *Cytometry B. Clin. Cytom.* **60,** 1–13 (2004).
3. Attar, A. Changes in the Cell Surface Markers During Normal Hematopoiesis: A Guide to Cell Isolation. *Glob. J. Hematol. Blood Transfus.* **1,** 20–28 (2014).
4. Manz, M. G., Miyamoto, T., Akashi, K. & Weissman, I. L. Prospective isolation of human clonogenic common myeloid progenitors. *Proc. Natl. Acad. Sci. U. S. A.* **99,** 11872–11877 (2002).
5. Majeti, R., Park, C. Y. & Weissman, I. L. Identification of a Hierarchy of Multipotent Hematopoietic Progenitors in Human Cord Blood. *Cell Stem Cell* **1,** 635–645 (2007).
6. Maecker, H. T., McCoy, J. P. & Nussenblatt, R. Standardizing immunophenotyping for the Human Immunology Project. *Nat. Rev. Immunol.* **12,** (2012).
7. Bendall, S. C. *et al.* Single-Cell Mass Cytometry of Differential Immune and Drug Responses Across a Human Hematopoietic Continuum. *Science (80-.).* **332,** 687–696 (2011).
8. Cserti-Gazdewich, C. M. *et al.* Quantitation of CD36 (platelet glycoprotein IV) expression on platelets and monocytes by flow cytometry: Application to the study of Plasmodium falciparum malaria. *Cytom. Part B - Clin. Cytom.* **76,** 127–134 (2009).
9. Nutt, S. L., Hodgkin, P. D., Tarlinton, D. M. & Corcoran, L. M. The generation of antibody-secreting plasma cells. *Nat. Rev. Immunol.* **15,** 160–171 (2015).
10. Juvet, S. C. & Zhang, L. Double negative regulatory T cells in transplantation and autoimmunity: Recent progress and future directions. *J. Mol. Cell Biol.* **4,** 48–58 (2012).
