# Supplementary material for: Parallel analysis of tri-molecular biosynthesis with cell identity and function in single cells
Source: Nat Commun. 2019 Mar 12;10:1185. doi: 10.1038/s41467-019-09128-7 (PMC6414513; doi:10.1038/s41467-019-09128-7)
Supplement: Supplementary file 3 — Reporting Summary [file 41467_2019_9128_MOESM3_ESM.pdf]

nature research

Corresponding author(s): Samuel C Kimmey, Luciene Borges, Reema Baskar, Sean C Bendall

Reporting Summary

Nature Research wishes to improve the reproducibility of the work that we publish. This form provides structure for consistency and transparency in reporting. For further information on Nature Research policies, see Authors & Referees and the Editorial Policy Checklist.

Statistical parameters

When statistical analyses are reported, confirm that the following items are present in the relevant location (e.g. figure legend, table legend, main text, or Methods section).

n/a	Confirmed	
☒	☐	The exact sample size (*n*) for each experimental group/condition, given as a discrete number and unit of measurement
☐	☒	An indication of whether measurements were taken from distinct samples or whether the same sample was measured repeatedly
☐	☒	The statistical test(s) used AND whether they are one- or two-sided<br>*Only common tests should be described solely by name; describe more complex techniques in the Methods section.*
☒	☐	A description of all covariates tested
☒	☐	A description of any assumptions or corrections, such as tests of normality and adjustment for multiple comparisons
☒	☐	A full description of the statistics including central tendency (e.g. means) or other basic estimates (e.g. regression coefficient) AND variation (e.g. standard deviation) or associated estimates of uncertainty (e.g. confidence intervals)
☒	☐	For null hypothesis testing, the test statistic (e.g. *F*, *t*, *r*) with confidence intervals, effect sizes, degrees of freedom and *P* value noted<br>*Give P values as exact values whenever suitable.*
☒	☐	For Bayesian analysis, information on the choice of priors and Markov chain Monte Carlo settings
☒	☐	For hierarchical and complex designs, identification of the appropriate level for tests and full reporting of outcomes
☒	☐	Estimates of effect sizes (e.g. Cohen's *d*, Pearson's *r*), indicating how they were calculated
☐	☒	Clearly defined error bars<br>*State explicitly what error bars represent (e.g. SD, SE, CI)*

Our web collection on statistics for biologists may be useful.

Software and code

Policy information about availability of computer code

Data collection	Single-cell mass-cytometry data was generated using a CyTOF2, with on-the-fly generation of single cell expression matrices as FCS files.
Data analysis	Single-cell mass-cytometry data was analyzed on cytobank.org, an on-line, cloud-based cytometry analysis platform. In addition, CYT, a matlab GUI created by the Dana Pe'er Lab, was used for developmental progression analysis. An R-script was created and used to generate the ordinary non-parametric bootstrapped median and confidence intervals for analyzed single cell bone marrow data, with utilization of the boot and flowCore R packages. A second R-script was created to implement FlowMap-FR analysis to statistically compare incorporation between bone marrow cell populations.

For manuscripts utilizing custom algorithms or software that are central to the research but not yet described in published literature, software must be made available to editors/reviewers upon request. We strongly encourage code deposition in a community repository (e.g. GitHub). See the Nature Research guidelines for submitting code & software for further information.

Data

Policy information about availability of data

All manuscripts must include a data availability statement. This statement should provide the following information, where applicable:

- Accession codes, unique identifiers, or web links for publicly available datasets
- A list of figures that have associated raw data
- A description of any restrictions on data availability

Single cell mass-cytometry datasets generated in this study will be publicly available at the time of publication. FCS files for HeLa cell cycle, whole blood and bone marrow experiments will be available, with filenames indicating the figure which data appears in and a brief description of sample condition.

Field-specific reporting

Please select the best fit for your research. If you are not sure, read the appropriate sections before making your selection.

☒ Life sciences ☐ Behavioural & social sciences ☐ Ecological, evolutionary & environmental sciences

For a reference copy of the document with all sections, see nature.com/authors/policies/ReportingSummary-flat.pdf

Life sciences study design

All studies must disclose on these points even when the disclosure is negative.

Sample size	Sufficient cell numbers from whole blood (>95,000 cells per sample) was collected in order to allow identification of major immune populations. Sufficient cell numbers from bone marrow donors (>325,000 cells per donor) were collected to allow identification of minor immature sub-populations including hematopoetic stem cells and progenitors, and minor mature sub-populations including plasma B-cells.
Data exclusions	Mass-cytometry data was pre-gated before analysis, explained further in the 'Gating strategy' of the Flow-Cytometry section. Single cell data used to generate the developmental trajectory of B-cell lymphopoesis was isolated from the entire bone marrow dataset. Bone marrow data from each donor was independently clustered based on the combined expression of 32 phenotypic parameters, and only cell clusters defined as hematopoetic stem cell, progenitor, and B-cell identities based on the expression of these 32 markers were subsequently used for developmental progression analysis.
Replication	SOM3B was validated on multiple cell lines including adherent and suspension lines. SOM3B was tested and validated on numerous peripheral blood samples from independent donors, providing reproducible results. Analyzed bone marrow samples from two independent donors validate observed characteristics across human hematopoiesis. Further, differing methods of mononuclear cell isolation and fixation provided similar results.
Randomization	Randomization was not relevant to the validation of SOM3B.
Blinding	Blinding was not relevant to the validation of SOM3B.

Reporting for specific materials, systems and methods

Materials & experimental systems

n/a	Involved in the study	
☒	☐	Unique biological materials
☐	☒	Antibodies
☐	☒	Eukaryotic cell lines
☒	☐	Palaeontology
☒	☐	Animals and other organisms
☒	☐	Human research participants

Methods

n/a	Involved in the study	
☒	☐	ChIP-seq
☐	☒	Flow cytometry
☒	☐	MRI-based neuroimaging

Antibodies

Antibodies used	A detailed table of all antibodies used in this work can be found in the supplementary tables, organized according to the experiments antibody reagents were used in. Information on manufacturer, clone, catalog number, and staining concentration are also included.

Validation	All newly reported metal-conjugated antibodies used in this study were extensively validated using positive and negative control cell lines and primary human peripheral mononuclear cells.

Eukaryotic cell lines

Policy information about cell lines

Cell line source(s)	Cell lines were sourced from ATCC.
Authentication	No authentication for cell lines was performed.
Mycoplasma contamination	No cell lines were tested for mycoplasma contamination.
Commonly misidentified lines (See ICLAC register)	HEK cells were used only to validate isotope-labeled anti-Puromycin antibody.

Flow Cytometry

Plots

Confirm that:

☒ The axis labels state the marker and fluorochrome used (e.g. CD4-FITC).

☒ The axis scales are clearly visible. Include numbers along axes only for bottom left plot of group (a 'group' is an analysis of identical markers).

☒ All plots are contour plots with outliers or pseudocolor plots.

☒ A numerical value for number of cells or percentage (with statistics) is provided.

Methodology

Sample preparation	Detailed sample preparation prior to antibody staining, staining procedures, and prep for CyTOF analysis is found in the detailed methods sections. Briefly, live cells in single cell-suspension were fixed with 1.6% PFA prior to long term storage in cell staining media (see detailed methods for formulation). All surface staining took place in 100 uL of antibody cocktail with CSM added to bring to volume, and occurred for 30 minutes at room temperature. Surface stained cells were washed with CSM and membrane permeabilized with ice cold methanol, on ice. Permeabilized cells then washed twice before intracellular antibody staining, carried out in the same manner as surface staining. After intracellular antibody staining, cells were washed and stored long-term in 1.6%PFA in PBS until data acquisition.
Instrument	CyTOF2 (mass-cytometry)
Software	Standard CyTOF2 instrument software was used during sample acquisition, and data then normalized against spiked in standardization beads using the matlab normalization GUI (created by Gary Nolan Lab), before subsequent analysis. Cytobank.org and CYT (matlab GUI created by Dana Pe'er group) was used to analyze single cell data.
Cell population abundance	No cells were sorted for this study.
Gating strategy	For all mass-cytometry data, sample data was first de-barcoded (inluding a cell-doublet removal step). Single cell data was further cleaned by removal of non-viable (viablility-dye positive) and cells actively undergoing apoptosis (postive stain for cleaved-Caspase3/cleaved-PARP). Detailed gating strategies used to isolate whole blood mononuclear cells in silico, and CD45positive bone marrow cells, are included in supplemental material.

☒ Tick this box to confirm that a figure exemplifying the gating strategy is provided in the Supplementary Information.
